# Helminth fauna of the monocled cobra (*Naja kaouthia*) from central Thailand: community composition and taxonomic perspectives

**DOI:** 10.1017/S0031182025100498

**Published:** 2025-08

**Authors:** Napat Ratnarathorn, Urusa Thaenkham, Abigail Hui En Chan, Panithi Laoungbua, Tanapong Tawan, Taksa Vasaruchapong, Vachirapong Charoennitiwat, Kittipong Chaisiri

**Affiliations:** 1 Applied Animal Science Laboratory, Department of Biology, Faculty of Science, Mahidol University, Bangkok, Thailand; 2 Laboratory of Helminth Biodiversity and Drug Development, Department of Helminthology, Faculty of Tropical Medicine, Mahidol University, Bangkok, Thailand; 3 Snake Farm, Queen Saovabha Memorial Institute, Bangkok, Thailand

**Keywords:** helminth community, helminth diversity, *Naja kaouthia*, parasite–host interactions, snake parasites

## Abstract

Snakes serve as important hosts for parasites at the interface of wildlife, humans and domestic animals. However, their helminth fauna in tropical regions, particularly Thailand, remains poorly documented. This study investigates the helminth fauna, community structure, infection patterns and their co-occurrence dynamics in the monocled cobra (*Naja kaouthia*) from urbanized areas of central Thailand, based on comprehensive analyses of 34 wild-caught individuals. Using integrated morphological and molecular approaches (e.g. mitochondrial 16S rRNA and *COI* gene sequencing), 11 helminth species were identified – 9 nematodes (*Kalicephalus bungari, Kalicephalus* sp. I and II, *Paracapillaria najae, Paracapillaria siamensis, Serpentirhabdias orientalis, Strongyloides* sp., a filarioid nematode and an unknown encysted nematode), 1 cestode (*Duthiersia expansa*) and 1 acanthocephalan (*Sphaerechinorhynchus* sp.) – including 2 newly described species: *P. siamensis* (infected in gastrointestinal tract) and *S. orientalis* (in respiratory tract). *Serpentirhabdias orientalis* and *K. bungari* were the most prevalent species (75.8%), while encysted nematodes exhibited the highest infection intensities (up to 500 parasites per host). Host–parasite network analysis revealed strong organ tropism in some helminth species (e.g. *Paracapillaria* spp.) and non-random co-occurrence patterns. Parasite abundance was positively correlated with host body condition, and females harboured greater species richness than males (11 vs. 8 taxa). The absence of trematodes is consistent with the host’s terrestrial ecology, while the low prevalence of filarioids (3.0%) suggests limited transmission. These findings underscore the value of integrative taxonomy in revealing hidden parasite diversity and highlight ecological drivers of infection patterns in snakes, with implications for wildlife health and zoonotic risk in human-modified landscapes.

## Graphical abstract

**Figure:**
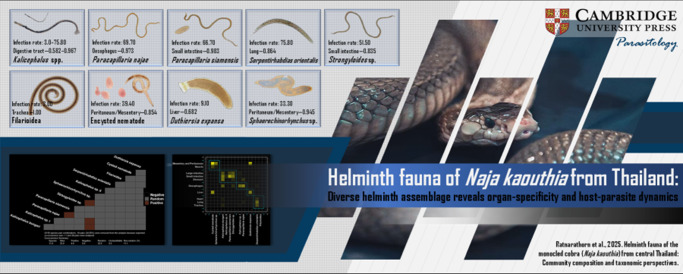

## Introduction

Rapid urbanization has intensified human–wildlife interactions, including those involving snakes, and has contributed to the growing popularity of exotic species introductions (Mendoza-Roldan et al., 2020; Ratnarathorn et al., 2024). Although snakes are inherently wild animals, several species have successfully adapted to anthropogenic environments by exploiting resources and seeking shelter within human communities (Yue et al., 2019; Hauptfleisch et al., 2021). This proximity increases the risk of pathogen transmission among snakes, humans and domestic animals.

In many cases, snakes encountered by humans are killed, though some species are consumed as food (Pandey et al., 2016; Babalola et al., 2020). In rural regions of Asia, including Thailand, the consumption of snakes as traditional food or medicine persists due to cultural beliefs associating snake-derived products with healing or aphrodisiac properties (da Nóbrega Alves et al., 2008; Vasaruchapong et al., 2017). However, these practices carry significant public health risks, as snakes can act as reservoirs of parasitic infections transmissible to humans, pets and livestock (Scientific opinion of the panel on biological hazards on a request from the European Commission, 2007; Hossain et al., 2023).

As carnivores and keystone predators, snakes occupy critical trophic positions within ecosystems and host a wide range of parasites (Mattison, 2007; Nature Conservancy, 2022). Their ecological role facilitates parasite transmission and supports the completion of complex life cycles (Davis et al., 2012; Mendoza-Roldan et al., 2020). Consequently, parasitic infections in snakes pose substantial threats to public health (Magnino et al., 2009; Tappe and Warrell, 2019; Bełcik et al., 2022), exotic pet management and domestic animal production systems (Tappe et al., 2011; Wolf et al., 2014; Saari et al., 2021; Charoennitiwat et al., 2024b). In wildlife populations, heavy parasitic burdens can result in morbidity and mortality (Santoro et al., 2013; Muangkaew, 2017; Miller et al., 2018; National Report, 2024). In addition, parasitized snakes may exhibit reduced venom yield, compromising antivenom production and increasing risks of the disease (Chaiyabutr and Chanhome, 2002).

Despite the ecological and medical importance of ophidian parasitology, research on snake parasites remains severely underrepresented relative to other vertebrate taxa (Fontenot and Font, 1996; Charoennitiwat et al., 2025). This knowledge gap is particularly notable in Southeast Asia, a region with high snake diversity, especially in Thailand (Cox et al., 2012; Hughes, 2017; Thai National Parks, 2022). Existing parasitological studies are limited in scope (e.g. Chaiyabutr and Chanhome, 2002; Vasaruchapong et al., 2017), and documented instances of snake-to-human parasitic transmission are rare (Anantaphruti et al., 2011; Boonyasiri et al., 2014). Expanding research in this field is essential for identifying emerging zoonotic threats and promoting safe practices in wildlife consumption and exotic animal care (Charoennitiwat et al., 2024a, 2025).

The helminth fauna of various cobra species has been extensively documented across Africa and Asia. These parasitological surveys have revealed a diverse array of nematodes, cestodes and trematodes inhabiting cobras worldwide. Among nematode parasites, ascarids are particularly significant. *Hexametra quadricornis* (Wedl, 1861) has been reported in African cobras such as *Naja nigricollis* Reinhardt, 1843, and *Naja melanoleuca* Hallowell, 1857 (Hering-Hagenbeck and Boomker, 2000). Another ascarid, *Ophidascaris najae* (Gedoelst, 1916), has been documented in numerous cobra species across both African and Asian continents, including *Naja haje* (Linnaeus, 1758), *Naja mossambica* Peters, 1854, *Naja naja* (Linnaeus, 1758), *Naja nivea* (Linnaeus, 1758), *Naja oxiana* (Eichwald, 1831), *Naja sputatrix* Boie, 1827, *Naja tripudians* Merrem, 1820, *N. nigricollis* and *N. melanoleuca* (Sprent, 1985; Farooq and Khan, 1994; Hering-Hagenbeck and Boomker, 2000). Strongylid nematodes of the genus *Kalicephalus* (Diaphanocephaloidea) have also been recorded in multiple cobra species, showing species-specific distribution patterns. These include *Kalicephalus bungari* (MacCallum, 1918) in *N. naja* from China and India, *Kalicephalus simus* (Daubney, 1923) in African cobras (*N. melanoleuca* and *N. mossambica*) and *Kalicephalus colubri* Ortlepp, 1923 in *N. melanoleuca* (Wang and Wang, 1992; Hering-Hagenbeck and Boomker, 2000). Other nematodes reported in cobras include the capillarid *Paracapillaria najae* (De, 1998) from *N. naja* in India (De, 1998) and the strongyloid *Serpentirhabdias fuscovenosa* (Railliet, 1899) in the African *N. nivea* (Hering-Hagenbeck and Boomker, 2000).

Platyhelminths have also been documented in cobra species. Cestode parasites reported include *Ophiotaenia indica* Johri, 1955 (Proteocephalidae) and *Oochoristica indica* Misra, 1945 (Davaineidae) from *N. naja* in Pakistan (Farooq and Khan, 1994). Trematodes include *Cyclorchis amphileucus* (Looss, 1896) Lühe, 1908 (Opisthorchiidae) in *N. haje* from Africa, and 3 Plagiorchiidae species – *Xenopharynx solus* Nicoll, 1912, *Encyclometra japonica* Yoshida and Ozaki, 1929, and *Encyclometra colubrimurorum* (Rudolphi, 1819) Dollfus, 1929 – reported from *N. naja* in Asia (Simha, 1964; Wang and Wang, 1992; Farooq and Khan, 1994). Despite the breadth of research on helminths in various cobra species, studies focusing specifically on the helminth fauna of the monocled cobra, *Naja kaouthia* Lesson, 1831, remain limited (but see Vasaruchapong et al., 2017; Charoennitiwat et al., 2023, 2024a, 2025).

The monocled cobra (*N. kaouthia*) is among the most frequently encountered snake species in Thailand (Jitakune, 2004; Wongtongkam et al., 2005; Ratnarathorn et al., 2024). It is a generalist predator that preys on a diverse range of animals, including small mammals, poultry, amphibians, reptiles and occasionally aquatic species such as eels (Chaitae, 2011). Its ecological adaptability, trophic versatility and frequent interaction with humans make *N. kaouthia* an ideal model for parasitological surveys. Furthermore, its use as a food source and known capacity to harbour multiple parasites highlight its relevance to both medical and wildlife management (Vasaruchapong et al., 2017).

This study aims to (1) identify parasite taxa infecting *Naja kaouthia* from central Thailand using morphological and molecular approaches, (2) characterize the parasitic helminth community structure, (3) provide insights into infection metrics of helminth assemblages and (4) analyse co-occurrence patterns and interactions among parasite taxa and between parasites and hosts. The findings aim to advance the field of wildlife parasitology and raise awareness of the zoonotic potential of helminth parasites shared among wild reptiles, humans and domestic animals.

## Materials and methods

### Host and parasite preparation

Thirty-four carcasses of the monocled cobra (*Naja kaouthia*) were obtained from the Snake Farm at Queen Saovabha Memorial Institute (Bangkok, Thailand). These specimens, initially rescued or captured by local villagers in Bangkok and adjacent provinces, included injured individuals that died during quarantine (2020–2023). All snakes were necropsied following standardized protocols (Ratnarathorn and Kongrit, 2024) to investigate helminth infections. Morphometric data (e.g. sex, weight, snout-vent length, tail length and scale counts) were recorded for species confirmation and host–parasite analyses.

After preservation at −20 °C, each specimen was dissected to obtain 11 internal organs: trachea, lung, heart and associated vessels, mesentery, gallbladder, liver, kidney, oesophagus, stomach, small intestine and large intestine. Organs were individually placed in Petri dishes containing one-quarter volume of tap water. The kidney and liver were compressed between glass plates and examined under stereomicroscopes (Olympus SZ30/SZ51, Japan); other organs were dissected and similarly screened. Body musculature and coelom were visually inspected, and any suspicious lesions were excised for microscopic evaluation.

Helminths and cysts were extracted using sterile micro-dissecting needles and precision probes, segregated by organ of origin, and transferred to water-filled Petri dishes. All specimens were rinsed with 0.85% physiological saline, fixed in 70% ethanol (stored in 1.5-mL cryotubes), and quantified. Processed samples and residual tissues were catalogued and archived at −20 °C in the Department of Helminthology, Faculty of Tropical Medicine, and the Department of Biology, Faculty of Science, Mahidol University, Thailand.

### Parasite identification

Helminth taxa were identified using an integrated approach that combined morphological and/or molecular techniques at the Department of Helminthology, Faculty of Tropical Medicine, Mahidol University. Taxonomic classification (Nematoda and Platyhelminthes) guided the application of standardized protocols for helminth specimen preparation and examination (Rojas et al., 2025). Morphological analyses were conducted using the stereomicroscopes, with diagnostic features compared against established references for species-level identification (e.g. Schad, 1962; Jones et al., 2005; Charoennitiwat et al., 2023, 2024a, 2025). Each specimen was documented with detailed morphological descriptions, morphometric data and, when applicable, molecular markers.

A few helminth species identified in this study had previously been described by the research team and published in peer-reviewed journals (i.e., Charoennitiwat et al., 2023, 2024a, 2025), while others are still undergoing detailed examination for species-level confirmation (see Table 1 for details). In this study, 1 adult filarioid nematode was identified solely based on morphological characteristics due to the limited specimen availability. Larval acanthocephalan was examined using a combined approach, integrating morphological traits as described by Schmidt and Kuntz (1996) and Amin (2013) with molecular data. In contrast, the encysted nematode and cestode were exclusively analysed using molecular techniques and are newly documented in this study.

**Table 1.** Verified prevalence and infection metrics of helminths in the monocled cobra, *Naja kaouthia* (*n* = 33) from Bangkok, Thailand

Helminth taxa [References]	Number of hosts infected	Infection rate (%) [95% CI]	MI [95% CI]	MA [95% CI]	Range
**Nematoda Family Diaphanocephalidae** *Kalicephalus bungari*	25	75.80 [57.69–88.26]	13.24 [8.40–20.08]	8.27 [6.12–15.85]	1–52
**Nematoda Family Diaphanocephalidae** *Kalicephalus* sp. I	12	36.40 [20.87–54.61]	14.25 [7.58−25.58]	5.18 [2.30−10.33]	1−48
**Nematoda Family Diaphanocephalidae** *Kalicephalus* sp. II	1	3.00 [0.16−16.11]	6.00 [NA]	0.18 [NA]	6
**Nematoda Family Capillariidae** *Paracapillaria najae* [Charoennitiwat et al., 2023]	23	69.70 [51.54−83.88]	16.78 [9.48−27.35]	11.70 [6.03−19.30]	1−60
**Nematoda Family Capillariidae** *Paracapillaria siamensis* [Charoennitiwat et al., 2024a]	22	66.70 [48.47−80.58]	29.05[16.64−55.77]	19.36 [10.03−40.09]	1−200
**Nematoda Family Rhabdiasidae** *Serpentirhabdias orientalis* [Charoennitiwat et al., 2025]	25	75.80 [57.69−88.26]	17.96 [8.48−47.16]	13.61 [6.33−39.18]	1−201
**Nematoda Family Strongyloididae** *Strongyloides* sp.	17	51.50 [34.69−68.41]	11.53 [7.35−18.82]	5.94 [3.36−10.67]	1−49
**Nematoda Family Filarioidea**	1	3.00 [0.16−16.11]	1.00 [NA]	0.03 [NA]	1
**Nematoda Superfamily Metastrongyloidea** Encysted nematode	13	39.40 [23.94−57.68]	124.08 [58.85−228.08]	48.88 [19.79−98.39]	1−419
**Cestoda Family Diphyllobothriidae** *Duthiersia expansa* (encysted)	3	9.10 [2.53−23.93]	289.33 [8.00−453.33]	26.30 [0.24−75.76]	8−500
**Acanthocephala Family Plagiorhynchidae** *Sphaerechinorhynchus* sp. (larvae)	11	33.30 [19.42−51.53]	3.64 [2.36−5.45]	1.21 [0.61−2.15]	1−9

MI, mean intensity; MA, mean abundance; 95% CI, 95% confidence interval.

For molecular analyses, genomic DNA was extracted from 1 to 6 helminth specimens using the DNeasy® Blood & Tissue Kit (Qiagen, Hilden, Germany), following the manufacturer’s protocol. Target genes included partial regions of the nuclear 16S ribosomal RNA (16S rRNA) for the encysted nematode and cestode, and the mitochondrial cytochrome c oxidase subunit I (*COI*) gene for the acanthocephalan. Taxon-specific primers and amplicon sizes used for the nematode and cestode (Chan et al., 2022), and for the acanthocephalan (Folmer et al., 1994), are presented in Table S2. Each 30 μL polymerase chain reaction (PCR) consisted of 15 μL of 2X i-Taq master mix (iNtRON Biotechnology, Gyeonggi, South Korea), 10 µM of each primer and 1 ng μL^−1^ of template DNA.

Polymerase chain reactions were performed using a T100™ thermocycler (Bio-Rad, California, USA) with thermal cycling conditions specific to each primer set, as shown in Table S2. PCR products were visualized using 1% agarose gel electrophoresis stained with SYBR Safe™ (Thermo Fisher Scientific, Massachusetts, USA). Selected amplicons were sent for sequencing using a fast next-generation sequencing platform (Tsingke, Beijing, China).

**Figure 1.**
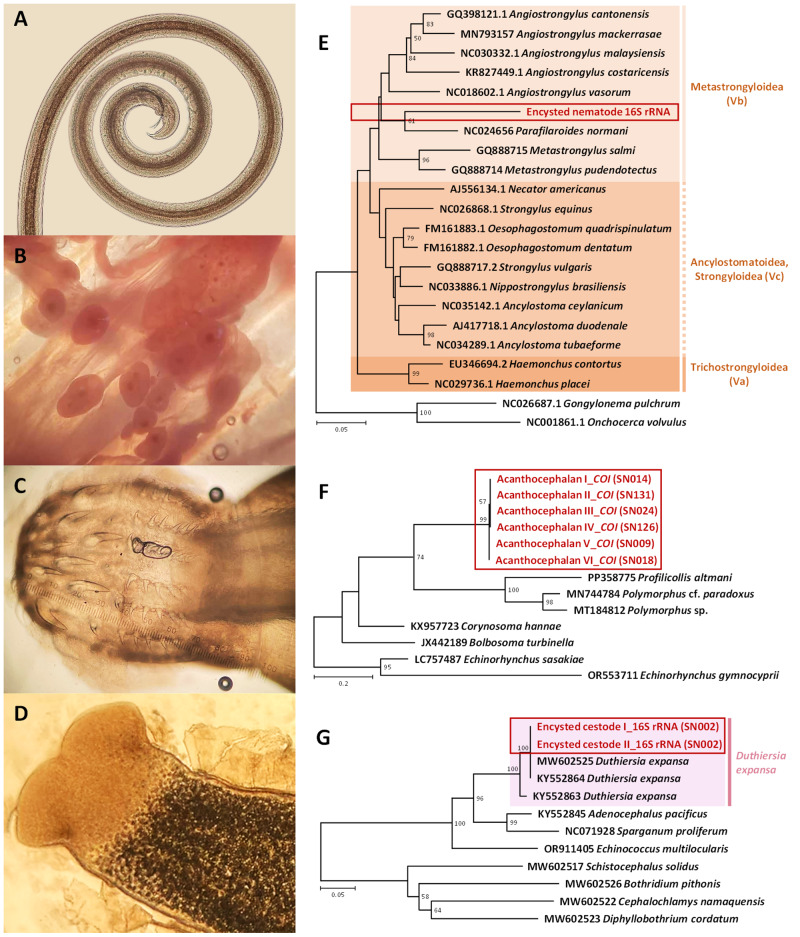
Morphological features and molecular phylogenetic placement of selected helminths recovered from *Naja kaouthia* in Thailand: (A) adult filarioid nematode showing a coiled, thread-like body; (B) encysted larval nematodes embedded in host tissue; (C) larval acanthocephalan with a bulbous proboscis bearing concentric rows of hooks; (D) larval cestode extracted from the cyst, displaying an invaginated scolex; (E) neighbour-joining phylogenetic tree of partial 16S rRNA sequences showing the placement of the encysted nematode (red box); (F) neighbour-joining phylogenetic tree based on *COI* gene sequences showing the acanthocephalan larvae (red box); and (G) neighbour-joining phylogenetic tree based on partial 16S rRNA sequences showing the encysted cestode (red box). Bootstrap values >50% are shown at nodes.

The obtained sequences were manually reviewed and edited using BioEdit version 7.2.5 (Hall, 1999). Phylogenetic trees were constructed based on neighbour-joining methods implemented in MEGA-11 (Tamura et al., 2021), with sequence alignments performed using ClustalX 2.1 (Thompson et al., 2002). Phylogenetic robustness was assessed using 1000 bootstrap replicates. All analyses incorporated available reference sequences from related taxa retrieved from GenBank (see Figure 1).

### Analysis of helminth infection and diversity in monocled cobras

Prevalence, mean abundance and mean intensity of infection with 95% confidence intervals (CIs) for each helminth taxon were estimated using Quantitative Parasitology 3.0 (Rózsa et al., 2000). Complete parasite count data are provided in the Supplementary Materials (Table S1). To examine the pairwise relationship between host body mass (weight-to-body length ratio) and the total abundance of helminth infection (log-transformed), a parametric Pearson’s correlation with a significance test was performed in R.

Helminth diversity estimation – including observed helminth species richness (HSR), species richness estimators (first-order Jackknife and Chao1), and the Shannon diversity index (H′) – was calculated across host variables (maturity, sex, and body mass groups) using the ‘BiodiversityR’ package (Kindt and Coe, 2005) in R software (R Core Team, 2025). HSR refers to the number of helminth species (or taxa) observed in a given host group, representing a direct count of species presence. The Jackknife (Jack1) and Chao1 estimators were used to provide more precise and less biased estimates of species richness and are considered particularly suitable for analysing parasite data compared to other estimators (Walther and Morand, 1998). The Shannon diversity index (H′) is widely employed to estimate diversity in ecological studies, including research on parasite communities (Ponlet et al., 2011; Shea et al., 2012; Chapman et al., 2015). In addition, helminth species accumulation curves were generated to assess the adequacy of cobra sample sizes and to visualize differences in HSR across host variables.

### Bipartite network analysis of host–parasite with target organ specificity

Host–parasite network analyses were conducted to investigate infection patterns across different host body systems. A bipartite network analysis was performed using an occurrence matrix (snake organs as rows and helminth species as columns), implemented via the ‘vegan’ (Oksanen et al., 2019) and ‘bipartite’ (Dormann et al., 2009) packages in R. The resulting network was visualized using the ‘computeModules’ function. The helminth species specificity index (ranging from 0 to 1) was calculated using the ‘specieslevel’ function to identify habitat specialists (species with limited organ distribution/fewer links) and habitat generalists (species with widespread organ distribution/ multiple links).

### Analysis of helminth species co-occurrence in the monocled cobra

Co-occurrence patterns among helminth species in monocled cobras were analysed using a probabilistic model implemented in the ‘cooccur’ package (Griffith et al., 2016) in R freeware (R Core Team, 2025). This model estimates the probability that helminth species pairs co-occur more or less frequently than expected by chance (Veech and Peres‐Neto, 2013). The analysis categorizes pairwise associations as positive, negative or random. Positive associations suggest that one parasite may facilitate the presence or transmission of another species, while negative associations may indicate interspecific competition or environmental constraints that prevent co-occurrence within the host.

## Results

### Helminth identification

Of the 34 *N. kaouthia* specimens examined, 33 (97.06%) were found to be infected with helminths. Analyses of these infected individuals revealed a diverse helminth community comprising at least 11 species: 9 nematodes, 1 acanthocephalan (*Sphaerechinorhynchus* sp.) and 1 cestode (*Duthiersia expansa* Perrier, 1873) (Table 1). Among the nematodes, 2 novel species – *Paracapillaria siamensis* Charoennitiwat, 2024 (Charoennitiwat et al., 2024a) and *Serpentirhabdias orientalis* Charoennitiwat, 2025 (Charoennitiwat et al., 2025) – have been formally described, while *Paracapillaria najae* represents a new host record for *N. kaouthia* in Thailand (Charoennitiwat et al., 2023). In addition, 1 *Strongyloides* species and 3 *Kalicephalus* species – one of which has been tentatively identified as *Kalicephalus bungari* – are currently under investigation for definitive species-level classification.

In this study, the identification of 3 helminth taxa required a combination of morphological and molecular approaches (Figure 1). A rare filarioid nematode was detected in 1 specimen and identified solely based on morphological characteristics due to the unavailability of additional material for DNA extraction. This nematode was characterized by its long, thread-like body, absence of a buccal capsule and sexually dimorphic features, including a coiled tail in male equipped with caudal alae and paired spicules (Figure 1A), consistent with descriptions of filarioids in Anderson (2000).

The larval acanthocephalan was morphologically assigned to the genus *Sphaerechinorhynchus*, exhibiting a bulbous proboscis with multiple concentric rows of hooks and a cylindrical body lacking a gastrointestinal tract (Figure 1C). These features were consistent with those described for *Sphaerechinorhynchus serpenticola* Schmidt and Kuntz, 1966, a species previously reported in *Naja naja* (Schmidt and Kuntz, 1996). Molecular analysis of the *COI* sequences did not provide species-level resolution; however, phylogenetic reconstruction placed the larval specimen within a clade containing *Polymorphus* and *Profilicollis* spp. (Figure 1F).

Two encysted helminths (a nematode and a cestode) could not be morphologically identified due to their larval stages (Figures 1B and 1D). Molecular identification classified the encysted nematode within the superfamily Metastrongyloidea (Figure 1E), clustering closely with *Parafilaroides normani* Dailey, 2009, although species-level assignment could not be confidently made. This finding suggests the presence of a metastrongyloid parasite in *N. kaouthia*.

In contrast, molecular techniques enabled confident species-level identification of the encysted cestode. Phylogenetic analysis placed the specimen within a strongly supported clade containing *Duthiersia expansa*, a diphyllobothrid cestode specific to reptiles (Figure 1G). Sequence data from the encysted individual confirmed its identity as *D. expansa* with maximal bootstrap support (100%), clearly separating it from closely related cestodes, e.g. *Adenocephalus pacificus* Nybelin, 1931, and *Sparganum proliferum* Ijima, 1905.

Overall, the integration of morphological examination and molecular analysis enabled the accurate identification of previously undocumented helminths in the monocled cobra, contributing new host and geographic records. The helminth community composition and species identifications are summarized in Table 1.

### Prevalence, infection intensity and abundance of helminth infection

Examination of 33 *Naja kaouthia* specimens revealed a diverse community of parasitic helminths, marked by variation in prevalence, infection intensity and mean abundance (Table 1). *Kalicephalus bungari* and *Serpentirhabdias orientalis* were the most prevalent species (75.8%, *n* = 25). The metacestodes of *Duthiersia expansa* revealed the highest mean intensity (MI = 289.33), while unidentified encysted nematode demonstrated the greatest mean abundance (MA = 48.88). These encysted nematodes (39.39% prevalence, MI = 124.08) occasionally reached extreme burdens, particularly within the mesentery. Likewise, *Duthiersia expansa* (9.09%) exhibited exceptionally high infection intensities (up to 500 samples/host), highlighting the heterogeneity and complexity of the helminth community in this cobra population. Two *Paracapillaria* species showed contrasting infection dynamics: *P. najae* exhibited broad host infection (69.70%) with moderate intensity (MI = 16.78), whereas *P. siamensis* had slightly lower prevalence (66.67%) but higher mean abundance (MA = 29.06). Less common taxa also contributed to the overall helminth diversity, including *Strongyloides* sp. (51.52%), *Sphaerechinorhynchus* sp. (33.33%) and *Kalicephalus* sp. I (30.30%). *Kalicephalus* sp. II was detected in only 1 individual (3.00% prevalence), with low infection intensity (MI = 6.00) and minimal abundance (MA = 0.18). Rare taxa such as the filarioid nematode (3.00%) further added to the richness of the helminth fauna, despite their low infection levels.

### Organ specificity of helminths

**Figure 2.**
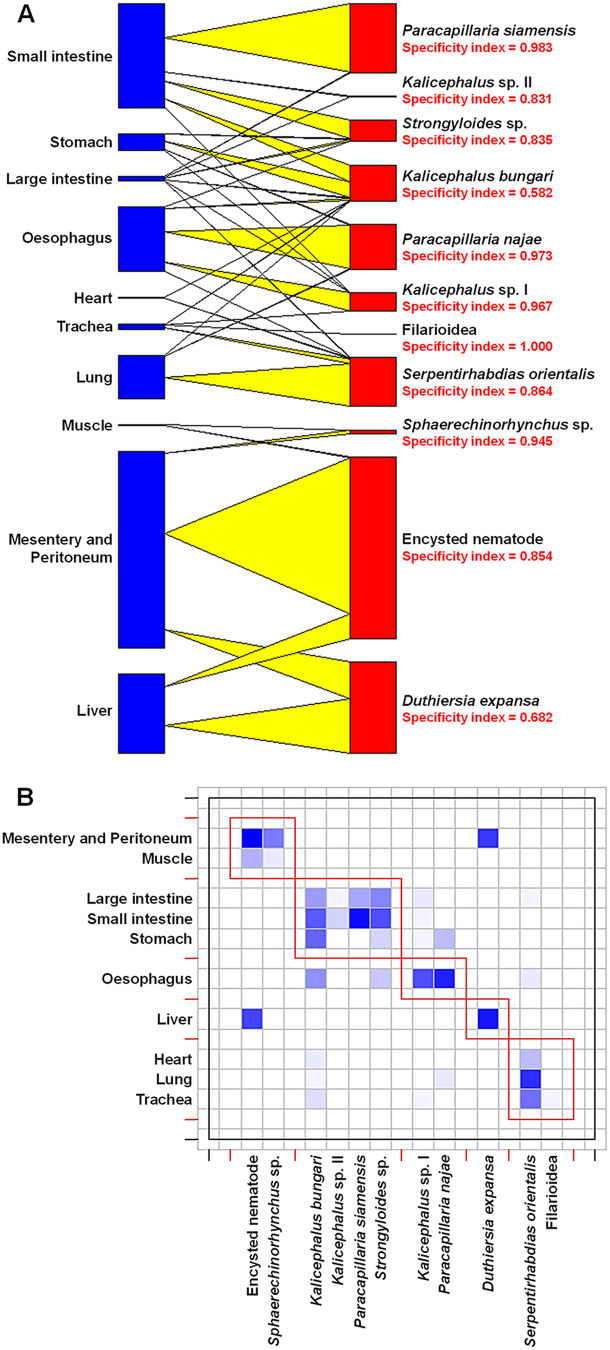
Organ specificity and distribution patterns of helminth parasites in *Naja kaouthia* hosts: (A) bipartite network illustrating organ specificity of 11 helminth species, with specificity indices ranging from 0 to 1 (where 1 indicates exclusive association with a single organ); and (B) schematic representation of helminth occurrences across anatomical sites, with darker shading indicating higher prevalence.

The helminth community in *N. kaouthia* exhibited strong niche partitioning across host organ systems, with distinct infection patterns revealed through network and specificity analyses (Figures 2A–B). *Paracapillaria* species demonstrated specialized tropism for upper gastrointestinal tissues (*P. najae*: oesophagus specificity = 0.973; *P. siamensis*: stomach = 0.983) while pulmonary systems were dominated by *S. orientalis* (trachea/lung = 0.864) and *Strongyloides* sp. (0.835) – though *S. orientalis* displayed life-stage segregation, with adults exclusively in respiratory organs but eggs in intestinal contents (not included in counts). Mesenteric and muscular tissues showed high specificity for acanthocephalans (*Sphaerechinorhynchus* sp. = 0.945) and encysted nematodes (0.854), contrasting with generalist *Kalicephalus* species that occupied multiple systems (indices 0.582–0.967).

### Host–parasite interactions and infection patterns

**Figure 3.**
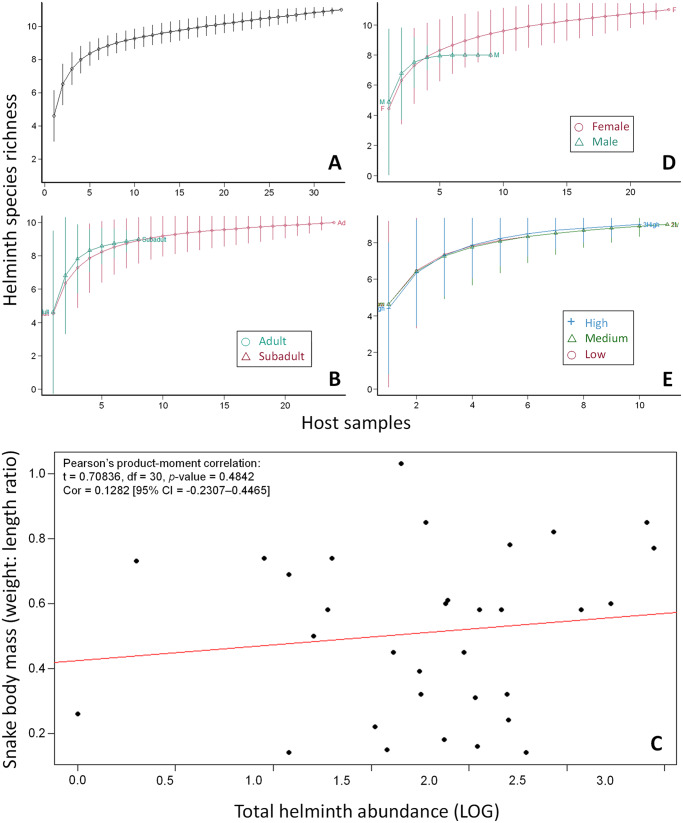
Variation in total helminth abundance across *Naja kaouthia* hosts in relation to sex, maturity, body condition and species richness. comparisons of helminth burden are presented for: (A) all hosts; (B) host maturity stages (adults vs. subadults); (D) sex (females vs. males); and (E) infection intensity categories (low, medium and high); (C) relationship between total helminth abundance and snake body condition, showing a significant positive correlation (Pearson’s *r*) between parasite burden and host body mass index.

Helminth community metric analyses in *N. kaouthia* revealed several significant patterns related to host characteristics (Figures 3A–E; Table 2). *Naja kaouthia* hosts harboured a core helminth community of approximately 11 species, with limited additional diversity detected beyond 20 specimens (Figure 3A), suggesting sample size adequacy for HSR estimation. Host maturity status did not significantly influence helminth community structure, with adults exhibiting somewhat higher species richness (10 species) compared to subadults (9 species) (Figure 3B). The results support the observation that larger sized *N. kaouthia* tended to harbour greater helminth loads, but not statistically significant (Pearson’s *r* = 0.1282, *P* = 0.4842; Figure 3C). While overall parasite abundance did not differ significantly between age classes (adults: 169.1; subadults: 87.4; Wilcoxon *p* = 0.7940), subadults exhibited more evenly distributed communities, as reflected by a higher Shannon index (1.88 vs. 1.82 in adults).

**Table 2.** Helminth community metrics by host maturity, sex and body mass

Metrics	*n*	Mean abundance [95% confidence interval]	Observed helminth species richness (HSR)	1st Jackknife estimator	Chao1 estimator	Shannon index (H‘)
**maturity**						
Adult	24	169.1 [66.5 to 271.7]	10	10.96	10.00	1.82
Subadult	8	87.4 [29.6 to 145.1]	9	9.88	9.00	1.88
**Sex**						
Female	23	157.7 [51.8 to 263.6]	11	12.91	12.00	1.79
Male	9	125.7 [37.3 to 214.1]	8	8.00	8.00	1.89
**Body mass**						
Low	11	85.9 [42.1 to 129.7]	9	9.91	9.45	1.94
Medium	11	140.5 [30.9 to 250]	9	9.91	9.45	1.39
High	11	226.8 [−13.8 to 467.4]	9	9.90	9.00	1.66

Sex-based differences were particularly notable (Figure 3D). Female snakes supported both greater parasite abundance (157.7 vs 125.7 in males) and higher species richness (11 vs 8 species), though these differences were not statistically significant (Wilcoxon *p* = 0.6598). The helminth diversity analysis revealed female-dominated infection patterns, with Jack1 (12.91 vs 8.00) and Chao1 (12.00 vs 8.00) estimators, suggesting greater estimated HSR in females.

Body mass effects of the hosts showed complex patterns (Figure 3E). While all mass groups shared identical observed richness (9 species), high-mass hosts exhibited substantially greater mean abundance (226.8) compared to low-mass individuals (85.9), though this trend was not significant (Kruskal–Wallis *p* = 0.8583). Low-mass hosts revealed superior community diversity (Shannon index: 1.94 vs 1.39–1.66 in other groups), indicating somewhat greater diversity despite lower overall abundance.

### Co-infection patterns in helminth community

**Figure 4.**
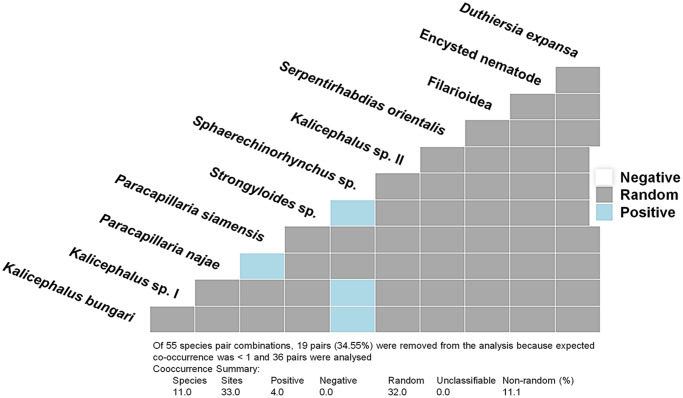
Co-occurrence matrix of helminth parasite species detected in *Naja kaouthia*, illustrating ecological associations among 11 helminth taxa. Each cell represents the interaction between a pair of species, categorized as positive (blue), negative (white) or random (grey).

Co-occurrence analysis revealed that helminth communities in monocled cobras are shaped by both random assembly processes and positive associations between specific species pairs (Figure 4). Of the 55 possible helminth species pair combinations, 36 were evaluated after excluding 19 pairs with low expected co-occurrence. Notably, 4 pairs exhibited significant positive associations (*Spaerethinorhynchus* sp. vs *Strongyloides* sp., *Spaerethinorhynchus* sp. vs *Kalicephalus* sp. I, *Spaerethinorhynchus* sp. vs *Kalicephalus bungari* and *Paracapillaria najae* vs *Paracapillaria siamensis*), while none showed negative associations – indicating a general tendency for co-infection rather than competitive exclusion. The high proportion of unclassified pairs (32 out of 36) likely reflects either neutral interactions or insufficient data for statistical resolution. The cystacanth larva of *Spaerethinorhynchus* sp. showed multiple positive associations with other helminths, suggesting that it may either be facilitated by, or itself facilitate, the presence of other parasites. The positive association between the 2 *Paracapillaria* species is also noteworthy, as it challenges the assumption that closely related species are prone to competitive exclusion. Overall, these findings underscore the complex structure of helminth communities in the monocled cobras, with *Spaerethinorhynchus* sp. potentially playing a central role in co-infection dynamics.

## Discussion

This study reveals a diverse and organ-specific helminth community in *N. kaouthia* from central Thailand, providing key insights into parasite diversity, host–parasite dynamics and broader ecological interactions. A total of 11 helminth taxa were identified, with nematodes dominating the assemblage, followed by an acanthocephalan (*Sphaerechinorhynchus* sp.) and a cestode (*Duthiersia expansa*). The observed patterns of helminth distribution across different host organs likely reflect species-specific tissue tropism driven by distinct life-history traits and physiological requirements, rather than active strategies to avoid interspecific competition. These results not only expand the known helminth diversity in Southeast Asian snakes but also offer ecological and evolutionary perspectives on parasite community structure in a key predator species.

The dominance of nematodes is likely associated with the cobra’s terrestrial ecology, which facilitates the nematode transmission while limiting exposure to trematodes. This limitation may stem not only from the ecological requirements of their intermediate hosts but also from the aquatic nature of the cercarial stage, which restricts transmission opportunities for hosts that are primarily terrestrial (Jones et al., 2005; Terrell and Stacy, 2007; Leung et al., 2019). Furthermore, the detection of adult stages in 8 out of 11 helminth species suggests that the monocled cobras in central Thailand serve as competent definitive hosts, supporting the completion of parasite life cycles within their environment and associated trophic networks. In contrast, the presence of larval and encysted forms may indicate potential for transmission to other trophic or paratenic hosts.

Although no confirmed human-specific pathogens were identified among the recovered helminths, the presence of several taxa raises concerns from both public health and ecological perspectives. Notably, the detection of larval and encysted nematodes highlights potential zoonotic risks, especially in regions where snakes are consumed as bushmeat (Vasaruchapong et al., 2017; Mendoza-Roldan et al., 2020). This concern is further supported by recent discussions on reptile-associated zoonoses, including those involving snakes, as outlined by Leung (2024). Exceptionally high parasite burdens – such as the 878 individuals recorded in the cobra specimen SN010 – could adversely affect host health, potentially compromising physiological functions, including venom production, which is critical for antivenom manufacturing (Chaiyabutr and Chanhome, 2002). These findings underscore the importance of continued monitoring, particularly as expanding urban landscapes increase the likelihood of human–snake interactions (Ratnarathorn et al., 2024).

The detection of *P. najae* (Charoennitiwat et al., 2023), *P. siamensis* (Charoennitiwat et al., 2024a) and *S. orientalis* (Charoennitiwat et al., 2025) represents significant additions to the regional helminth fauna and underscores the understudied diversity of ophidian parasites in Thailand and the broader South-east Asian region. These discoveries underscore the importance of comprehensive parasitological surveys, especially as anthropogenic pressures, climate change and habitat loss intensify ecosystem disruptions (e.g. Brooks and Hoberg, 2007; Lettoof et al., 2019). Although these species are not known to infect humans, their identification enhances understanding of tropical snake host–parasite networks and provides a baseline for monitoring shifts in parasite distributions (e.g. Oliveira et al., 2024). Documenting this diversity is crucial for predicting ecological consequences and mitigating emerging wildlife–human interactions.

The high prevalence of *Kalicephalus* spp., particularly *K. bungari* (75.8%), which predominantly inhabits the digestive tract, is consistent with previous reports of its frequent occurrence among Southeast Asian snakes (Chaiyabutr and Chanhome, 2002), reinforcing its status as a common regional parasite. However, the earlier record by Chaiyabutr and Chanhome (2002) identified the species as *Kalicephalus laticaudae* Yamaguti, 1935, based on limited evidence likely resulting from taxonomic misinterpretation. In this study, organ-specific partitioning, along with preliminary molecular and morphological analyses, suggests the presence of at least 3 distinct *Kalicephalus* species infecting *N. kaouthia*, emphasising the significance of these hookworms as key components of the cobra’s helminth community. This finding aligns with the observations of Vasaruchapong et al. (2017), who also reported *Kalicephalus* species as one of the dominant nematodes in Thai cobras. In contrast, the low prevalence of filarioid nematodes (3.0%) may reflect sporadic transmission dynamics, low infectivity or host resistance (e.g. Davis et al., 2012; Mendoza-Roldan et al., 2020). The infrequent detection of *Kalicephalus* sp. II and the presence of unresolved species complexes further highlight the need for expanded sampling efforts and molecular characterization to clarify taxonomic ambiguities and accurately document helminth diversity.

The results of this study align with and expand upon the earlier survey by Vasaruchapong et al. (2017), which reported a lower helminth diversity (6 taxa) in *N. kaouthia* from central Thailand, predominantly consisting of nematodes such as rhabditids and *Kalicephalus* spp. In contrast, this study identified a more diverse helminth community (11 species), including newly described taxa such as *P. siamensis* and *S. orientalis*. This increased diversity likely reflects methodological improvements, including the incorporation of molecular techniques and organ-level dissections. Notably, Vasaruchapong et al. (2017) detected only rhabditid eggs in the gastrointestinal tract without recovering adult specimens, likely due to limitations in observational methods. Both their study and the present one report a near absence of trematodes. Rather than being solely attributable to the terrestrial lifestyle of *N. kaouthia*, this absence is more plausibly explained by dietary habits – specifically, the lack of consumption of suitable second intermediate hosts such as semi-aquatic invertebrates (e.g. insects), which are required for trematode transmission (Leung et al., 2019; Leung, 2024). Furthermore, the network analysis employed in the present study revealed clear niche partitioning among helminths (e.g. *Paracapillaria* spp. in the digestive tract), a dimension not previously explored. These findings underscore the importance of methodological advancements in parasite detection and the role of ecological factors in shaping helminth assemblages.

Host characteristics also appear to influence helminth community structure. A positive correlation (but not statistically significant) was observed between host body condition (body mass index) and parasite abundance, potentially reflecting greater foraging effort and increased exposure to infected intermediate hosts in healthier individuals (Lopez and Duffy, 2021). Female cobras harboured more helminth species (11) than males (8), a pattern that may be driven by ecological and physiological factors (Morand and Hugot, 1998). Ecologically, females are typically larger than males in many snake species, which allows them to consume more and larger prey items that may serve as intermediate hosts for helminths (Shine, 1994). This broader dietary range increases their likelihood of exposure to parasitic infections. Physiologically, sex-based differences in hormonal immunity may also contribute: testosterone in males is generally immunosuppressive, while oestrogens in females can enhance certain immune functions (Zuk and McKean, 1996; Klein, 2000). This may promote tolerance rather than parasite clearance, enabling females to host a broader range of helminths without experiencing severe pathology, thereby contributing to the observed species richness. Behavioural factors such as differential habitat use or increased feeding effort in gravid females may also elevate exposure to infected intermediate hosts (Roberts et al., 2004). The observed trend (not statistically significant) contrasts with findings from other host–parasite systems (e.g. Poulin, 1996; Moore and Wilson, 2002), warranting further investigation into the drivers of sex-biased parasitism in snakes.

Organ-specificity analyses demonstrated strong tropism among helminth taxa. *Paracapillaria* spp. were dominantly localized in the digestive system, while *S. orientalis* showed a strict association with the respiratory system. This spatial partitioning likely minimizes interspecific competition and reflects niche specialization (Dunne et al., 2002). Co-occurrence analysis identified 4 positively associated species pairs and no negative associations, suggesting facilitative interactions or shared transmission pathways. These finding imply facilitative relationships rather than competitive exclusion (Holmes, 1987; Poulin, 2001). Several mechanisms may underlie these associations, including shared microhabitat preferences within host organs, overlapping transmission routes involving similar intermediate hosts, host environment modification by 1 species to creates favourable conditions for another, and phylogenetic constraints influencing host exploitation strategies (Sukhdeo and Bansemir, 1996; Poulin and Morand, 2000; Lello et al., 2004).

Notably, positive associations were observed between congeneric species (*P. siamensis* and *P. najae*) and between taxonomically distinct groups (e.g. *Spaerethinorhynchus* sp. and the 2 *Kalicephalus* spp.), suggesting that phylogenetic relatedness may influence parasite community assembly patterns. Furthermore, the facilitative relationship between the 2 *Paracapillaria* spp. challenges the traditional assumption that closely related parasites necessarily compete for similar resources. Instead, niche partitioning or resource specialization may promote coexistence even among phylogenetically similar taxa (Friggens and Brown, 2005; Rynkiewicz et al., 2015). However, empirical data from this study indicate a tendency towards mutual exclusion between *P. najae* and *P. siamensis*, with the former predominantly inhabiting the oesophagus and the latter confined to the intestinal tract. This microhabitat partitioning possibly reduces competition for attachment sites and food resources.

Despite the valuable insights obtained, several limitations warrant consideration. The sample size (*n* = 34) may have restricted the detection of rare taxa and reduced statistical power in co-occurrence analyses. Additionally, the presence of morphologically ambiguous or cryptic species, particularly among *Kalicephalus*, underscores the importance of integrating molecular tools in future studies (Thaenkham et al., 2022). Long-term monitoring and life cycle elucidation are essential to better understand transmission dynamics and the ecological roles of these helminths within snake populations and broader ecosystems.

In conclusion, this study provides the first comprehensive characterization of helminth communities in *N. kaouthia* from central Thailand, revealing a diverse assemblage dominated by nematodes with significant organ specificity and host–parasite dynamics. The high prevalence of certain helminth species (e.g. *K. bungari*) and the discovery of novel taxa (*P. siamensis* and *S. orientalis*) underscore the understudied parasite diversity in Southeast Asian snakes. Although statistically insignificant, the positive correlation between host body condition and parasite abundance, along with female-biased species richness, suggests ecological and physiological drivers of infection patterns. While no direct zoonotic threats were identified, the presence of larval and encysted forms highlights potential risks in regions where snakes are consumed. These findings emphasize the need for integrative (morphological and molecular) approaches in wildlife parasitology and long-term monitoring to assess the impacts of urbanization on parasite transmission. Future studies should explore the life cycles of rare taxa (e.g. filarioids) and the immunological mechanisms underlying observed host–parasite patterns.

## Supporting information

Ratnarathorn et al. supplementary material 1 Ratnarathorn et al. supplementary material

Ratnarathorn et al. supplementary material 2 Ratnarathorn et al. supplementary material

## Data Availability

The data that support the findings of this study are available from the first and corresponding authors upon reasonable request.

## Supplementary materials

The supplementary material for this article can be found at https://doi.org/10.1017/S0031182025100498.
