# Supplementary material for: Helminth fauna of the monocled cobra (Naja kaouthia) from central Thailand: community composition and taxonomic perspectives
Source: Parasitology. 2025 Jul 14;152(9):938–50. doi: 10.1017/S0031182025100498 (PMC12644949; doi:10.1017/S0031182025100498)
Supplement: Ratnarathorn et al. supplementary material 2 — Ratnarathorn et al. supplementary material [file S0031182025100498sup002.pdf]

**Table S2:** Taxon-specific primers, thermal cycling conditions, and amplification details.

| Taxon | Gene | Primer | Primer sequence | Length (bp) | Denaturation | Extension | Reference |
|---|---|---|---|---|---|---|---|
| Nematode | 16S rRNA | 16S-nematodeC345-F<br>16S-nematodeC345-R | 5′-AAGATAAGTCTTYGGAARYT-3′<br>5′-GAAYTAAACTAATATCAMG-3′ | ~240 (partial) | 94 °C_5 min, 35 cycles of 94 °C_30 sec, 45 °C_45 sec 72 °C_1 min | 72 °C_5 min | Chan *et al.*, 2022 |
| Cestode | 16S rRNA | 16S-platyhelminth-F<br>16S-platyhelminth-R | 5′-GTGYDAAGGTAGSATAAT-3′<br>5′-CCGGTYT YAACTCARCTCAT-3′ | ~379 (partial) | 94 °C_5 min, 35 cycles of 94 °C_30 sec, 56 °C_45 sec 72 °C_1 min | 72 °C_5 min | Chan *et al.*, 2022 |
| Acanthocephalan | *COI* | LCO<br>HCO | 5′-GGTCAAC AAATCATAAAGATATTGGT-3′<br>5′-TAAACTTCAGGGTGACCAAAAAATCA-3′ | ~721 (partial) | 94 °C_5 min, 35 cycles of 94 °C_1 min, 50 °C_1 min, 72 °C_1 min | 72 °C_10 min | Folmer *et al.*, 1994 |
